# Predictive modeling of parafoveal information processing during reading

**DOI:** 10.1038/s41598-021-92140-z

**Published:** 2021-06-21

**Authors:** Stefan Seelig, Sarah Risse, Ralf Engbert

**Affiliations:** 1 Department of Psychology, University of Potsdam, 14469 Potsdam, Germany; 2 Research Focus Cognitive Sciences, University of Potsdam, 14469 Potsdam, Germany

**Keywords:** Human behaviour, Dynamical systems

## Abstract

Skilled reading requires information processing of the fixated and the not-yet-fixated words to generate precise control of gaze. Over the last 30 years, experimental research provided evidence that word processing is distributed across the perceptual span, which permits recognition of the fixated (foveal) word as well as preview of parafoveal words to the right of fixation. However, theoretical models have been unable to differentiate the specific influences of foveal and parafoveal information on saccade control. Here we show how parafoveal word difficulty modulates spatial and temporal control of gaze in a computational model to reproduce experimental results. In a fully Bayesian framework, we estimated model parameters for different models of parafoveal processing and carried out large-scale predictive simulations and model comparisons for a gaze-contingent reading experiment. We conclude that mathematical modeling of data from gaze-contingent experiments permits the precise identification of pathways from parafoveal information processing to gaze control, uncovering potential mechanisms underlying the parafoveal contribution to eye-movement control.

## Introduction

High-acuity visual processing is limited to the center of the visual field (the fovea) with an extension of about 2$^\circ$, which fits a short word at typical font size and stimulus distance. Consequently, humans need to generate fast eye movements (saccades) to move words into the fovea for word recognition during natural reading^1^. However, the visual field is much larger than that and words are processed, although with lower visual acuity, beyond the fovea (in the parafovea). Here, we report results on the use of word information from the parafovea for eye-movement control during reading. We present an explicit computational model of parafoveal processing in an experimental paradigm. Our approach is fully predictive, i.e., the model is trained under natural reading conditions and makes predictions for the effects of experimental manipulations of the reading process.

A critical concept for information processing during reading is denoted as the perceptual span^2^, which is the area of the visual field in which text must be visible for the reader to proceed reading at a normal speed. Experimentally, the perceptual span has been measured by systematically increasing the size of a window of visible text that moves with the readers’ gaze across the sentence until readers cease to show significant disruption in their reading behavior^2–4^. The average size of the perceptual span extends roughly from 3 to 4 letters to the left of fixation to about 14–15 letters to the right of fixation and is therefore asymmetric around the fixation location^5^. However, low-level pre-attentive processes such as crowding^6^ also modulate visual processing, so that the letter identification span is effectively up to 7–9 letters to the right of fixation^7^. Nevertheless, the concept of the perceptual span is strongly associated with word recognition processes and, therefore, with the allocation of attention during reading.

### Experimental findings on parafoveal processing

**Figure 1:**
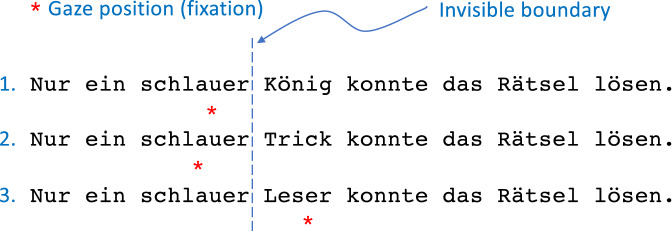
The boundary technique as a variant of gaze-contingent displays. The critical word position is to the right of an invisible boundary. If gaze position is to the left of the boundary (*first line*), the preview word is diplayed. The preview is either a high-frequency word (König) or a low-frequency word (Trick). A saccade crossing the boundary triggers an immediate display change that replaces the preview by a medium-frequency word (Leser).

The boundary paradigm^2^ is among the most frequently used experimental methods to study the effects of parafoveal processing on the timing of the reader’s eye movements during reading. Contingent on the reader’s gaze position, the parafoveal preview of a target word (word $n+1$) is manipulated in an *invalid preview* condition (e.g., a random letter string or a different word is presented), while the reader’s eyes fixate to the left of it (e.g., before or on the pretarget word *n*). When a saccade is launched past the location of an invisible boundary placed after the last letter of word *n*, the preview of word $n+1$ is changed and replaced by the target word (Fig. 1). Readers typically lack awareness of such display changes^8–12^. Conversely, in the *valid preview* condition the preview is identical to the target word, thus representing normal reading.

In experiments using the boundary paradigm, readers show differences in fixation durations as a function of the preview condition in which the sentence was presented. The first finding in the boundary paradigm is an effect of the preview validity in fixation durations on the target word $n+1$ to the right of the boundary. Fixation durations on word $n+1$ are longer when an invalid preview was presented and shorter when the valid (identical) word was displayed before its fixation^13^. This difference in fixation durations is typically interpreted as a preview benefit resulting from a headstart of processing the identical preview in parafoveal vision^14^. As parafoveal preprocessing reduces the word’s remaining processing demand, word recognition times are shorter when the word is finally fixated.

The second finding is an effect of the preview difficulty. Fixation durations are longer when the parafoveal preview was a difficult word (high processing load) and shorter when it was an easy word (low processing load). Manipulating the parafoveal processing load by the preview’s lexical frequency (i.e., the frequency of occurrence of a word in a representative text database), preview difficulty effects have not been observed on the pretarget word *n* before the boundary^15,16^ but on the target word $n+1$ after the boundary^10,16–18^. While it seems clear that the preview must have been preprocessed up to its lexical level in parafoveal vision, the precise mechanisms that prolong the critical fixation after the boundary can be investigated using explicit computational models.

### Computational predictions for eye-movement control

Several computational models^19^ of eye-movement control have been developed over the last 20 years. Interestingly, many model comparisons are limited to qualitative analyses so far^20^, mainly due to the lack of adequate statistical methods for model inference of complex process-oriented cognitive models^21^. The SWIFT model^22^ provides a conceptually convenient architecture in the context of implementing mechanisms for the contributions of foveal and parafoveal processing on eye-movement control; the model provides a platform for studying interactions between foveal and parafoveal processing without major changes of the model principles.

Another prerequisite for the investigation of quantitative predictions is a reliable framework for statistical inference. Recently, we implemented a fully Bayesian framework for parameter inference for the SWIFT model^23,24^, which permits parameter identification based on experimental data from single readers in a statistically rigorous way. Therefore, we implement our assumptions on the interaction of fixation duration with foveal and parafoveal processing in the SWIFT model to investigate the potential of various mechanisms in explaining the integration of foveal and parafoveal information during reading.

**Figure 2:**
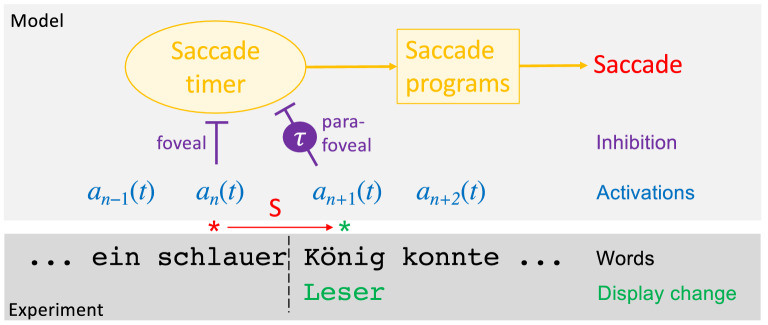
Modeling eye-movement control in the boundary paradigm. Experiment: the saccade S triggers the display change from the preview König to the fixated word Leser. Model: Word-based activations indicate states of lexical processing for each word. The saccade timer initiates a cascade of processes that produce the saccade. Inhibition of the saccade timer can delay the saccade, which is observed as increased fixation duration. Based on model simulations, we investigated whether inhibition by foveal (word *n*) alone or with parafoveal words (word $n+1$) is more consistent with experimental data. Parafoveal inhibition can be immediate ($\tau =0$) or delayed ($\tau >0$).

In the SWIFT model^22^ fixation durations are controlled by a random saccade timer that initiates new saccade programs, which accounts for the stochasticity in fixation durations (see Supplementary Note 1). Influences from cognitive word processing are introduced into the model by inhibitory processes. Each word *n* is represented in SWIFT by an activation $a_n(t)$ at time *t* under the assumption of parallel processing^25^. Processing difficulties for low-frequency words produce higher lexical activations on average which delay the start of the upcoming saccade program. Consequently, fixations on difficult words show increased fixation durations (Fig. 2). In the lastest version of SWIFT^23,24^, only the processing of the currently fixated word in the fovea affects the random timer through foveal inhibition. In this study, we investigate additional parafoveal inhibition from activation $a_{n+1}(t)$ to the right of the fixated word *n*. In two different variants, parafoveal inhibition can act on the timer either immediately ($\tau =0$) or with temporal delay ($\tau >0$). The temporal delay assumption in the model permits a test for how increased lateral masking in peripheral vision^26^ affects the time course of lexical processing in foveal and parafoveal vision^27^. The display change was implemented as a reset of the target word’s activation values to zero, and would restart processing with the first fixation after the boundary during invalid preview conditions.

For our simulation studies, we adopted a fully predictive framework, where the model was fitted to data of the control condition only (i.e., with valid preview), while data of two invalid preview conditions, (1) invalid high-frequency (HF) preview or (2) invalid low-frequency (LF) preview, were simulated as quantitative predictions. The difference between the mean fixation durations of the invalid HF and LF preview conditions estimated the preview difficulty effect, whereas the difference between valid and invalid preview conditions (the latter computed as the average of mean fixation durations in HF and LF conditions, respectively) tested the preview validity effect. The model simulations of the boundary experiment provide a strong test of possible pathways from foveal and parafoveal information to gaze control within a well-defined mathematical model under statistically reliable procedures^21,23,24^.

Previous simulations in the boundary paradigm^28^ have shown that the SWIFT model can account for the preview benefit of word $n+2$ (one word further into the right parafovea than word $n+1$) by its principle of foveal inhibition. However, this mechanism alone could not satisfactorily explain the additional effect of preview difficulty. Parafoveal difficulties may inhibit oculomotor control similarly as foveal difficulty^10^ making it a purely cognitive effect. Alternatively, the effect may be an oculomotor consequence of the boundary paradigm itself by inducing saccadic inhibition^29^ when the display change occurs. While empirical observations support the first^16^, a process-defined comparative test with simulations is still outstanding.

In a first step, we therefore explored to what extent cognitive control mechanisms could suffice to account for the spatio-temporal pattern of preview effects in the boundary paradigm (i.e., no preview difficulty and validity effects on word *n* but on word $n+1$). Consequently, we extended the model’s cognitive control from only foveal (P0) to also parafoveal inhibition. Parafoveal inhibition was either acting immediately (P1) or with a delay of 100 ms (P2) accounting for the slower processing efficiency in parafoveal vision. After estimating model parameters, we determined the mean prediction errors of these three model variants.

In a second step, we further analyzed possible interactions between properties of the experimental method and the oculomotor control system. Therefore, we simulated two different types of saccade cancelation scenarios in response to the display change in the invalid preview conditions, similar to saccadic inhibition^29^. The first scenario assumed that a substantial proportion of saccade programs with a probability of $p=0.5$ is canceled based on the visual disruption when replacing the invalid preview with the target word. Such a mechanism has successfully accounted for prolonged fixation durations in scene onset delay experiments^30^. The second scenario assumed that the successful cancelation further depends on the stage of preview processing and is more likely when the preview is still in the phase of increasing lexical activations. This interaction of visual and lexical pathways might be a necessary assumption to account for the asymmetric display-change effects with respect to word frequency.

## Results

For the boundary experiment (see “Experimental data” section), we carried out numerical simulations to generate predictions of the SWIFT model with foveal (P0), parafoveal (P1), and time-delayed parafoveal (P2) inhibition. We also investigated three assumptions on possible saccade cancelations due to display changes, i.e., without cancelation (baseline), with saccade cancelation (SC), and with cancelation limited to saccades during the increasing stage of lexical activation (SC-L1). In sum, we investigated nine different models. Since we focus on lexical parafoveal processing in an $n+1$ boundary paradigm, we restricted our analyses of fixation durations to fixation sequences where a single fixation on word *n* was followed by a first fixation on word $n+1$. Sequences with multiple fixations on word *n* were excluded from analysis in simulated as well as experimental data.

Model parameters were estimated for each participant and model based on the experimental control condition data (see “Bayesian parameter inference” section). Posterior predictive checks were carried out to ensure successful parameter estimations (see “Evaluation of parameter estimations” section). Since our approach was to predict the outcome of the experimental boundary manipulations, parameter estimates from the control condition were used for the experimental conditions (see “Implementation of the experimental paradigm and model variants” section).

### Fixation durations on the post-boundary word

**Figure 3:**
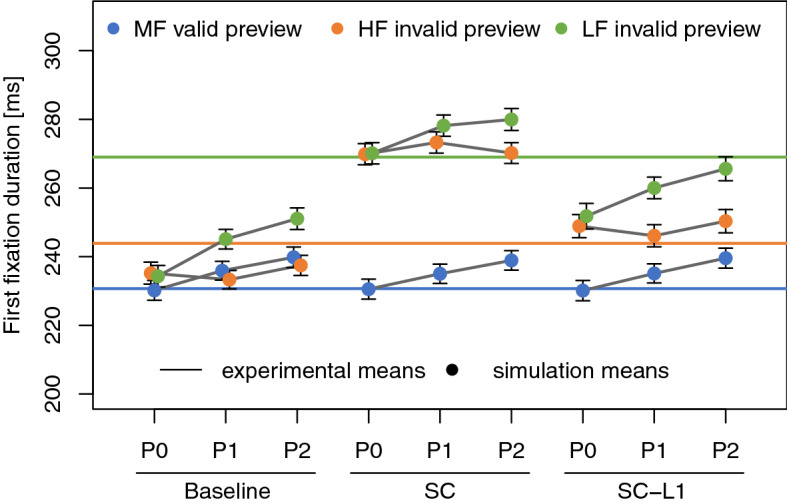
Model comparison of $n+1$ fixation durations as a function of three preview conditions. Horizontal lines reflect the empirical condition means using the same color legend. P0: Foveal inhibition only. P1: Foveal and parafoveal inhibition. P2: Foveal and delayed parafoveal inhibition. Baseline: Simple processing reset after display change. SC: Additional saccade cancelation. SC-L1: Saccade cancelation during lexical processing (L1) only.

Simulations of nine model variants generated predictions of the reading behavior in two preview conditions based on parameters fitted to the control condition with valid preview. We evaluated the first fixation duration on the post-boundary word (Fig. 3, Table 1) after single fixations on word *n*. Models only incorporating inhibition by foveal processing (P0) predict, on average, the same fixation durations after the boundary when changing from an easy (HF) preview to the target word as compared to when changing from a difficult (LF) preview to the target word. When parafoveal processing difficulties within the processing span were inhibiting the autonomous saccade timer (P1), a difference between HF and LF invalid preview condition emerged and the mean values of the simulated fixation durations became larger when a difficult LF preview was processed in parafoveal vision. The effect of a delay of 100 ms of the inhibiting effect of the parafoveal information (P2) is less obvious and our simulations indicate that the effects of the delay unfold in interaction with saccade cancelations.

**Figure 4:**
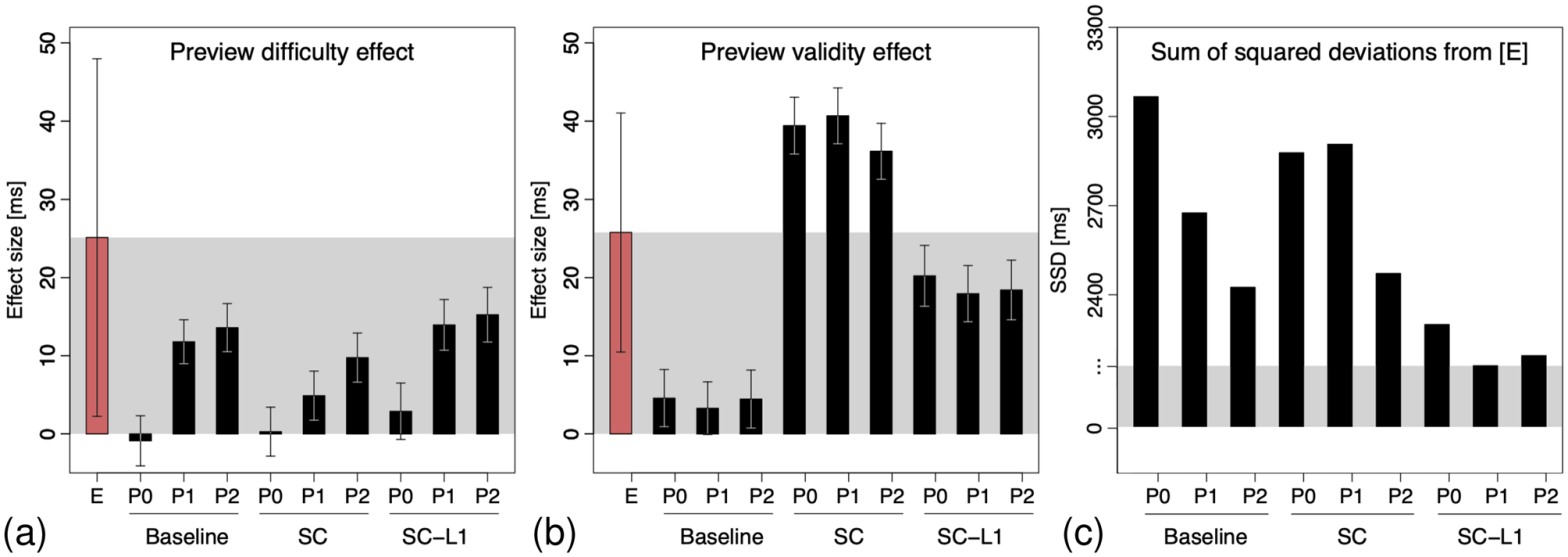
Predicted parafoveal preview effects for the first fixation after the boundary. (**a**) Preview difficulty effect. Smallest difference to experimental effects is observed for baseline models P1/P2 and SC-L1 model P1/P2. (**b**) Preview validity effect. Smallest difference from experimental effects obtained for SC-L1 models. (**c**) Overall model scoring. The mean sums of squared deviations from experimental effect sizes indicate best performances for models that combine parafoveal inhibition with processing dependent saccade cancelation.

The three baseline model variants failed to show a substantial benefit of processing a valid (identical) preview in parafoveal vision as compared to an invalid preview. Only after implementing saccade cancelations (SC) based on the display change in the two invalid preview conditions, the condition means differed and a preview validity effect was observed (see also Fig. 4b). Saccade cancelation further seemed to interact with the parafoveal inhibition mechanisms. In the saccade cancelation models (SC), the mean difference between HF and LF invalid fixation durations in the presence of immediate parafoveal inhibition (P0) was smaller than if parafoveal inhibtion was delayed (P1). In the baseline models (without saccade cancelation) and the processing-dependent saccade-cancelation models (SC-L1), however, this difference was almost of the same size and did not differ much between the two parafoveal inhibition variants.

### Predicted preview effects

All of the nine model variants reproduce important summary statistics with respect to word frequency and word length (Table 2, see also Fig. S3). With respect to the spatio-temporal pattern of parafoveal preview effects in the boundary paradigm, results are summarized in Fig. 4 and Table 1. We can conclude that parafoveal inhibition is crucial for the model to account for the novel preview difficulty effect in $n+1$ fixations after the boundary (P1 and P2 models). However, to account for the classical preview validity effect, the present simulations required additional mechanisms. Implementing the latter as a purely cognitive effect via foveal and parafoveal inhibition of saccadic programming only showed small preview validity effects. Thus, the preview validity effect is best explained by saccade cancelations after a display change with only a small portion of cognitive preprocessing benefits (i.e., 4–6 ms reflected in the preview validity effect of the baseline models, Fig. 4b). Moreover, the cognitive preview benefit portion is already fully developed in the P0 baseline model (i.e., no further increase in the P1 and P2 baseline models). Thus, in contrast to the preview difficulty effect, the preview validity effect is fully accounted for by foveal inhibition and does not require further parafoveal inhibition. At the qualitative and quantitative level, the models P1 and P2 with processing-dependent saccade cancelations (SC-L1) show both the best account of preview difficulty and preview validity effects in fixation $n+1$ after the boundary (Fig. 4c).

**Table 1:** Mean first fixation durations on word $n+1$ and preview effect sizes in the experiment and model simulations, with $95\%$ confidence intervals in parentheses.

	E	Baseline			SC			SC-L1		
		P0	P1	P2	P0	P1	P2	P0	P1	P2
**First fixation durations by condition**										
HF	$243.9\,(13.6)$	$235.2\,(3.2)$	$233.3\,(2.7)$	$237.5\,(2.9)$	$269.8\,(3.1)$	$273.3\,(3.1)$	$270.2\,(3.0)$	$248.9\,(3.4)$	$246.1\,(3.2)$	$250.4\,(3.4)$
MF	$230.7\,(10.0)$	$230.2\,(2.9)$	$235.9\,(2.7)$	$239.8\,(3.0)$	$230.6\,(2.9)$	$235.0\,(2.8)$	$238.9\,(2.8)$	$230.1\,(3.0)$	$235.1\,(2.8)$	$239.6\,(2.9)$
LF	$269.0\,(18.0)$	$234.3\,(3.1)$	$245.1\,(2.9)$	$251.1\,(3.2)$	$270.1\,(3.1)$	$278.2\,(3.1)$	$280.0\,(3.2)$	$251.8\,(3.7)$	$260.0\,(3.2)$	$265.6\,(3.5)$
**Preview effects**										
Difficulty	$25.1\,(22.9)$	$-\,0.9\,(3.2)$	$11.8\,(3.2)$	$13.6\,(3.2)$	$0.3\,(3.1)$	$4.9\,(3.1)$	$9.8\,(3.1)$	$2.9\,(3.6)$	$13.9\,(3.6)$	$15.2\,(3.6)$
Validity	$25.8\,(15.3)$	$4.6\,(3.7)$	$3.3\,(3.7)$	$4.5\,(3.7)$	$39.4\,(3.6)$	$40.7\,(3.6)$	$36.1\,(3.6)$	$20.2\,(3.9)$	$17.9\,(3.9)$	$18.4\,(3.9)$
mSSD	–	3066.4	2675.3	2424.9	2877.7	2906.5	2471.2	2299.7	2160.6	2194.9

CIs are smaller in simulations due to the increased data volume. The models were scored using the mean sum of squared deviations (mSSD) of simulated preview effects from experimental results per participant.

**Table 2:** Effects of word frequency (LF–HF) and word length (long–short) for various measures of fixation durations and fixation probabilities for experimental and model simulations data of the control condition (MF). These summary statistics refer to the control condition without boundary manipulation, i.e., different model variants with saccade cancelation do not apply here.

	Word frequency				Word length			
	Exp	P0	P1	P2	Exp	P0	P1	P2
**Probability**								
Skipping	$-.16$*	$-.20$*	$-.20$*	$-.20$*	.23*	.26*	.25*	.25*
Single fixation	$-\,.08$*	$-\,.16$*	$-\,.16$*	$-\,.16$*	.07*	.19*	.19*	.19*
Regression (out)	.02	.02*	.02*	.01*	$-\,.01$	$-\,.02$*	$-\,.02$*	$-\,.02$*
Refixation	.08*	.16*	.16*	.16*	$-\,.07$*	$-\,.19$*	$-\,.19$*	$-\,.19$*
**Duration**								
1st fixation duration	$-\,1.83$	12.55*	16.57*	16.25*	$-\,13.94$	$-\,14.36$*	$-\,18.19$*	$-\,16.42$*
2nd fixation duration	$-\,4.62$	$-\,2.82$	$-\,5.82$*	1.15	$-\,9.68$	2.92	9.39*	$-\,.45$
Single fixation duration	14.13*	8.31*	13.60*	14.99*	$-\,11.50$	$-\,7.36$*	$-\,9.67$*	$-\,12.16$*
Refixation duration	$-\,4.62$	$-\,2.82$	$-\,5.82$*	1.15	$-\,9.68$	2.92	9.39*	$-\,.45$
Gaze duration	27.53*	41.05*	45.86*	46.84*	$-\,27.14$*	$-\,4.30$*	$-\,42.42$*	-44.71*
Total viewing time	43.04*	55.59*	59.23*	58.47*	$-\,43.30$*	$-\,55.19$*	$-\,56.40$*	$-\,57.28$*

Frequency and length classes (from 2nd word to word n-1 in the sentence including target word) were determined via median split. Asterisks indicate differences, where $p<.05$ (Welch’s *t* test).

## Discussion

In the current study we investigated mechanisms for a dynamical model of eye-movement control during reading that can account for preview effects which appear in two flavors, the preview validity and preview difficulty effect. We used data from an experiment^16^ with an $n+1$ boundary paradigm and three different preview difficulties to estimate posterior distributions of parameters in the SWIFT model of eye movements during reading^22^. Parameters were estimated independently for three different implementations of cognitive influences on fixation durations: Inhibition of saccade programming by foveal processing only (P0), additional inhibition by parafoveal processing (P1), and delayed parafoveal inhibition (P2). Estimations were restricted to data from the valid preview condition. Based on the obtained posterior distributions over the model parameters, we predicted fixation sequences for all experimental conditions in the estimated model variants. Each model variant was further crossed with three implementations of the effects of the display change occurring in boundary paradigms. In the baseline models, word processing was simply restarted after the display change, whereas in the two saccade cancelation models the display change could also impede saccade programming either generally, or coupled to the lexical stage of word processing (for a detailed motivation of each model see the “Introduction” section). From the simulated data we calculated the effect sizes for the preview validity effect and the preview difficulty effect for the first fixation after the boundary.

The baseline model P0 with only foveal inhibition did not yield preview difficulty effects, but it was sufficient to elicit a small effect of preview validity. The effect is brought about by the reset of activation of the target word at the onset of its first fixation during invalid preview conditions. While during valid conditions processing of the target word is already in an advanced stage and completed soon after the fixation onset, during invalid conditions processing must start over, resulting in a longer period during which foveal inhibition can influence the random saccade timer. The reason why this influence was small as compared to previous simulations with the SWIFT model^28^ is the different dynamics in the $n+2$ as compared to the $n+1$ boundary paradigm. Generally, once the random saccade timer has initiated a saccade program that later elicits a gaze shift, foveal inhibition can no longer affect the current fixation duration. Foveal inhibition, therefore, needs to be fast and strong enough already at the beginning of fixation to affect the first fixation on the target word. In the present simulations, the reset of lexical activation occurred just at the moment of the first fixation on the target word and foveal inhibition was, on average, rather low. In the $n+2$ boundary paradigm, in contrast, the reset had often happened before the target word was fixated leading to relatively higher foveal inhibition.

The introduction of parafoveal inhibition in model P1 did bring about a preview difficulty effect through the mechanism described above. When the model fixates word *n* and has initiated a saccade program to word $n+1$, inhibition resulting from the parafoveal preview affects the duration of the upcoming fixation, therefore increasing durations of the upcoming fixation on the target word $n+1$ in case of LF previews, as compared to HF previews. Delaying the influence of parafoveal information by 100 ms in the P2 models shifts the evolution of activation more consistently into the time window where the saccade timer’s activity is related to the fixation duration on the target word. This affects fixation durations in LF conditions more than in HF conditions, which likely is the result from a dynamical interaction of word frequency with the increase in fixation duration itself.

Display change induced saccade cancelation was introduced as a mechanism to reflect the disruption of visual stimulus continuity that occurs in the case of a display change. Here, if a labile saccade stage is active during the display change, it is aborted with a fixed probability of 50%. A new labile stage can then be initiated in the regular way by the main saccade timer. Unlike with regular saccade cancelation, where an ongoing labile stage is canceled and immediately replaced by a new one (initiated by the main timer), in display change induced saccade cancelation the labile stage is aborted without an immediate replacement. This substantially increases some fixation durations after the display change (i.e., 50% of them) and induces a strong validity effect (see Fig. 4).

The third model type was used to investigate processing-dependent saccade cancelation. Psycholinguistic theory suggests that word processing can often be approximated by a two stage process and consists of lexical and post-lexical stages. Research indicates that the visually presented word stimulus is more important during lexical processing^31^ (whereas post-lexical integration can proceed even when the visual representation is absent), which should be reflected in the model. Hence, the display change sensitive saccade cancelation was coupled to the word processing stages within SWIFT, where the epoch of rising activation represents the lexical processing (L1), and the epoch of falling activation represents post-lexical processing^22^ (L2). In these model variants (going by SC-L1) labile stages can only be canceled if word processing was still in the earlier L1 stage (although activation reset was done for every invalid preview on crossing the boundary, irrespective of the stage of word processing). This reduced the size of the effect of saccade cancelation on the mean fixation duration in invalid preview conditions, resulting in a pattern more aligned with the data observed in the experiment.

In our simulation study, we used the SWIFT model as a platform for the different variants of parafoveal processing. The parallel processing framework is an open architecture for testing effects of distributed processing^25^. Working within a parallel framework does not automatically reproduce the experimentally observed preview effects, of course. On a qualitative level, because of its architecture, the SWIFT model potentially generates a range of different preview effect. However, all model behavior is controlled by model parameters, which regulate the effect size and the presence and absence of effects. The precise numerical values of the model parameters must be selected objectively by statistical inference. We published a related rigorous framework for parameter inference in dynamical models^23,24^, which is applied in the current study as well. As a consequence, while the SWIFT model might produce a certain preview effect in general, the fit to given experimental data in combination with objective parameter inference, can still fail to reproduce this preview effect (see^28^ for an example). From this perspective, the study of the current model variants investigates their specific contributions to the explanation of preview effects for given experimental data under an objective statistical inference framework (e.g., Bayesian dynamical modeling^21^).

For the future perspective of the current work, it should be noted that the recently published OB1-Reader model^32^ proposed how letter-level visual and lexical processing could be successfully integrated into a model of eye-movement control. Based on such extensions, we expect that even more specific predictions of eye-movement behavior in the boundary paradigm and its variants will be possible.

Finally, it is important to stress that the results presented in this work heavily rely on the success and quality of the parameter estimation. Parameter inference based on individual readers’ experimental data might be a breakthrough for process-oriented modeling^21,23,24^, since interindividual differences are often comparable in size to the observed effects. Here we exploited the full potential of interindividual differences by running predictive simulations separately for each participants and showed that preview effects in the boundary paradigm are due to an integration of foveal and parafoveal processing demands. While the preview difficulty effect is best accounted for by delayed inhibition of the readers gaze due to parafoveal lexical activation, the classical preview validity effect relies on inhibition of foveal word activation only, boosted by saccade program cancelation after display changes. This simulation study suggests that the preview difficulty effect is determined by cognitive, rather than by oculomotor processes, a finding in line with interpretations of previous empirical observations^10,16^ and a potential benchmark for other reading models.

## Methods

### Experimental data

Experimental data were collected in an experiment of single sentence reading with 34 participants^16^. Each participant read 114 sentences on a computer monitor in a single session, while their eyes were being tracked. The experiment used the gaze contingent boundary paradigm, where an invisible boundary is placed between two adjacent words. In the beginning of a trial the word displayed to the right of the boundary corresponded to one of three different preview conditions. Those preview words had the same word length but could either be of high frequency (HF), medium frequency (MF) or low frequency (LF). Then, as soon as the eyes first crossed the boundary towards the preview, the preview was replaced by a target word of medium frequency. In the MF condition the preview and the target word were identical. The process of replacing the word on the monitor was implemented to be quick enough, that the saccadic movement which had triggered the boundary would envelope the display change event. Of the 3521 fixation sequences in the collected data, only fixation sequences in the MF condition were selected for parameter estimation. Sequences containing less than three fixations or fixations longer than one second were not considered in the estimation. Additionally, all fixations after regressions from the last or second to last word were removed. This left 1139 fixation sequences with a total number of 10,172 fixations from 34 participants.

### Bayesian parameter inference

The parameter estimations were conducted using a Python implementation^33^ of the $\hbox {DREAM}_{ZS}$ algorithm^34^ from the class of Metropolis-Hastings Markov chain Monte Carlo (MH-MCMC) algorithms^35^. In a Bayesian framework MH-MCMC algorithms use a random walk strategy to iteratively build up a sampling distribution which eventually converges to the posterior distribution $P(\theta |y)$ of parameters $\theta$ given the data *y*. Starting with the chains randomly dispersed in parameter space $\Theta$, the sampler generates new proposals from perturbations of the latest positions of the chains at each iteration. The proposals are integrated into the chain depending on their acceptance probability.

For each of the three models, parameter estimations were conducted using three chains per participant, with 20, 000 iterations per chain. As priors for the parameters, we used Gaussian distributions truncated at one standard deviation, with ranges according to Table S1. Since calculating the likelihood in SWIFT uses simulations and approximations, whereby the likelihood is inflated with a stochastic error^23^, the $\hbox {DREAM}_{ZS}$ algorithm had to be slightly modified. A stochastic likelihood can have adverse effects on the algorithm’s rate of convergence. Originally, at any given position of a chain the likelihood is evaluated only once^34^. However, stochastic fluctuations of likelihood values can impede the calculation of the acceptance ratio and introduce long periods of stagnation in the evolution of chains where no proposals are accepted. To circumvent this, the modified algorithm newly evaluates the likelihood for the latest chain position at every iteration. While this doubles the computational costs, it also prevents the algorithm from becoming stuck. As a result, we obtained the posterior over the set of model parameters (see Figure S1)^23^.

Numerical calculations were carried out on the high-performance computing cluster of the Norddeutscher Verbund für Hoch- und Höchstleistungsrechnen (HLRN). Parallel computation was used at the level of the estimation algorithm, as well as the level of trials within each participant-wise model evaluation, respectively.

### Implementation of the experimental paradigm and model variants

The experimental manipulation involved a display change event where, during invalid conditions, an invalid word preview is replaced with a target word. In simulations of the valid MF condition, no changes in model architecture had to be made, since this condition represented normal reading. In the HF and LF conditions the word frequency of the word $n+1$ corresponded to the respective invalid previews by the beginning of a trial. Once the model had finished the execution of a saccade past the last letter of word *n*, the frequency of word $n+1$ was changed to represent the MF word. Additionally, the activation values of the target word were reset to zero and the processing stage was reset to the first stage.

Parafoveal inhibition (P1) was implemented in a similar fashion as the existing mechanism for foveal inhibition^23^. The numerical values of the word activations were multiplied with their respective inhibition factors (see Table S2), before modulating the transition rate of the saccade timer. The delay was implemented using a memory array, where activations of parafoveal words were retained together with their time signature, so they could be recalled after the delay of 100 ms.

Two variants of the model implement saccade cancelation in the invalid preview conditions as a result of the display change. In SWIFT, the two-stage process of saccade programming can only be aborted during the first, labile stage, but not during the non-labile, second stage. In the event of a successful cancelation, a new saccade program is immediately initiated, starting with a labile stage, thereby increasing the duration of the current fixation. The causes of cancelations are now extended to include display change events. When a labile stage is active during the time of the display change in the SC model, it is canceled with a probability of $p=0.5$. In the SC-L1 model it is also required that the second processing stage of the preview word $n+1$ has not yet been reached.

Artificial fixation sequences were simulated for all subjects with parameter combinations specific to the subjects’ estimation results. For each sentence a different set of parameters was randomly sampled from the posterior distribution of the respective participant^24^, and per sentence 10 sequences were generated. For the analysis of preview effects, the simulated sentences were processed in the same way, as the experimental data. To keep results comparable, parameters were sampled from the posterior distributions once per participant and sentence. One set of parameters was estimated for each participant and model variant P0, P1, and P2. Each set of parameters was then used in all simulations for the respective participant in the Baseline, SC, and SC-L1 models and all conditions for the same sentences. Simulations of three participants were excluded from all further analyses due to computer error.

### Evaluation of parameter estimations

For a first analysis of the effects of the specific model implementation on the parameter estimation, based on the posterior distributions we calculated the estimation mean of the subjects median parameter values, to compare them with the lower and higher margins of $30\%$ highest posterior density intervals (HPDIs) of the posteriors pooled over participants (Table S1). We observe that variability between estimations is lowest for parameters related to spatial aspects of oculomotor control, and higher for parameters concerned with temporal control of saccade timing and word processing.

Posterior predictive checks^36^ were done for the three sets of parameter estimations P0, P1 and P2. Each set consists of 31 distinct simulations based on the posterior distributions of individual participants. The posterior distributions correspond to the experimental control condition (MF). We calculated a set of common summary statistics (Table S3, Figure S2) from the data of all experimental conditions and the simulated data, in order to cross validate the model fit. Significant Pearson correlations coefficients indicate good agreement of experimental and simulated data in the HF and LF conditions, which were not used in the parameter estimations.

## Supplementary information

Supplementary Information.

## Data Availability

The experimental data used in this study were published before^16^ and were made publicly available via the Open Science Framework (DOI 10.17605/OSF.IO/KZ483). Simulated data are accessible with the source code of the model (see below).

## Code availability

Source code used for numerical simulations, analyses, and plotting as well as other project-related files are made available via the Open Science Framework (DOI 10.17605/OSF.IO/gdsn7/).
