# Supplementary material for: Predictive modeling of parafoveal information processing during reading
Source: Sci Rep. 2021 Jun 21;11:12954. doi: 10.1038/s41598-021-92140-z (PMC8217250; doi:10.1038/s41598-021-92140-z)
Supplement: Supplementary file 1 — Supplementary Information. [file 41598_2021_92140_MOESM1_ESM.pdf]

Supplementary Information:
Predictive modeling of parafoveal information processing during reading

Stefan Seelig, Sarah Risse, & Ralf Engbert
University of Potsdam, Department of Psychology, Potsdam, 14469, Germany

Supplementary Contents

This file includes:

- Supplementary Note 1 (Mathematical details of the SWIFT model)
- Figure S1 (Posterior densities from simulations)
- Figure S2 (Scatter plot of simulated and experimental measures)
- Table S1 (Results from parameter estimation)
- Table S2 (Inhibition parameters in model variants)
- Table S3 (Correlations between simulated and experimental measures)

Supplementary Note 1 (Mathematical details of the SWIFT model)

In the SWIFT model[22] we proposed two independent mechanisms for target selection and saccade timing, coupling via word-based activations. Word activations represent the current state of word processing over time. At the same time, word activations control target selection for an upcoming saccade and modulate fixation durations via delay of upcoming saccades. The internal state of the model[23] at time t can be written as $n = (n_1, n_2, ..., n_{4+N_W})$ with $n_1, ..., n_4$ representing saccade timers and $n_5, ...n_{4+N_W}$ word activations, where the total number of words in a given sentence is denoted by N_W. Word activations increase during lexical processing and decrease during postlexical processing. All random variables n_i are discrete, so SWIFT is a continuous-time, discrete state random walk model (which can be simulated efficiently via its master equation[23]).

Within a processing span centered at the current gaze location, words are processed in parallel[25]. The eccentricity of letter j in word i is given by $\varepsilon_{ij}(t)$, which is time-dependent due to changes of gaze position via saccades. The spatial extension of the processing span with δ letter spaces to the left and to the right of fixation is a very important parameter. We assume an inverse parabolic processing function, which is the dependence of the processing rate $\lambda(\varepsilon)$ from eccentricity ε, i.e.,

$$\lambda(\varepsilon) = \lambda_0 \cdot \left\{ \begin{array}{lll} 1 - \varepsilon^2/\delta^2, & \text{for} & |\delta| \leq \varepsilon \\ 0, & \text{otherwise} & \end{array} \right. , \tag{1}$$

where $\lambda_0 = 3/4\delta$ is a normalization constant. The processing rate $\Lambda_i(t)$ for word i at time t is given as

$$\Lambda_i(t) = L_i^{-\eta} \sum_{j=1}^{L_i} \lambda(\varepsilon_{ij}(t)) , \tag{2}$$

with word length L_i and parameter η as an exponent determining the influence of word-length.

A word's activation increases with rate $\Lambda_i(t)$ during lexical processing. When the word-frequency dependent maximum is reached, post-lexical processing starts with a decrease in activation determined by the same processing rate. Additionally, there is a decay rate ω accounting for memory leakage effect[24]. Saccade target selection is a stochastic process with targeting probability $\pi(m,t)$ for word m at time t controlled by relative activation, i.e.,

$$\pi(m,t) = \frac{a_m(t)}{\sum_{j=1}^{N_W} a_j(t)} . \tag{3}$$

Finally, saccades are generated with random inter-saccade intervals[23]. To account for word difficulty effects, random timing is inhibited by foveal activation. Thus, the rise-rate of the saccade timer is modulated by a factor $(1 + h a_k(t))^{-1}$, where $a_k(t)$ is the foveal (fixated) word k, so that high activation delays an upcoming saccade and, therefore, prolongs ongoing fixation duration. In Figure 2, we illustrate how the concept of foveal inhibition is generalized to investigate influences of parafoveal processing; in this case inhibition acts by slowing factor $(1 + h a_{k+1}(t))^{-1}$ from word $k + 1$. Correspondingly, delayed parafoveal inhibition is given by a factor $(1 + h a_{k+1}(t - \tau))^{-1}$.

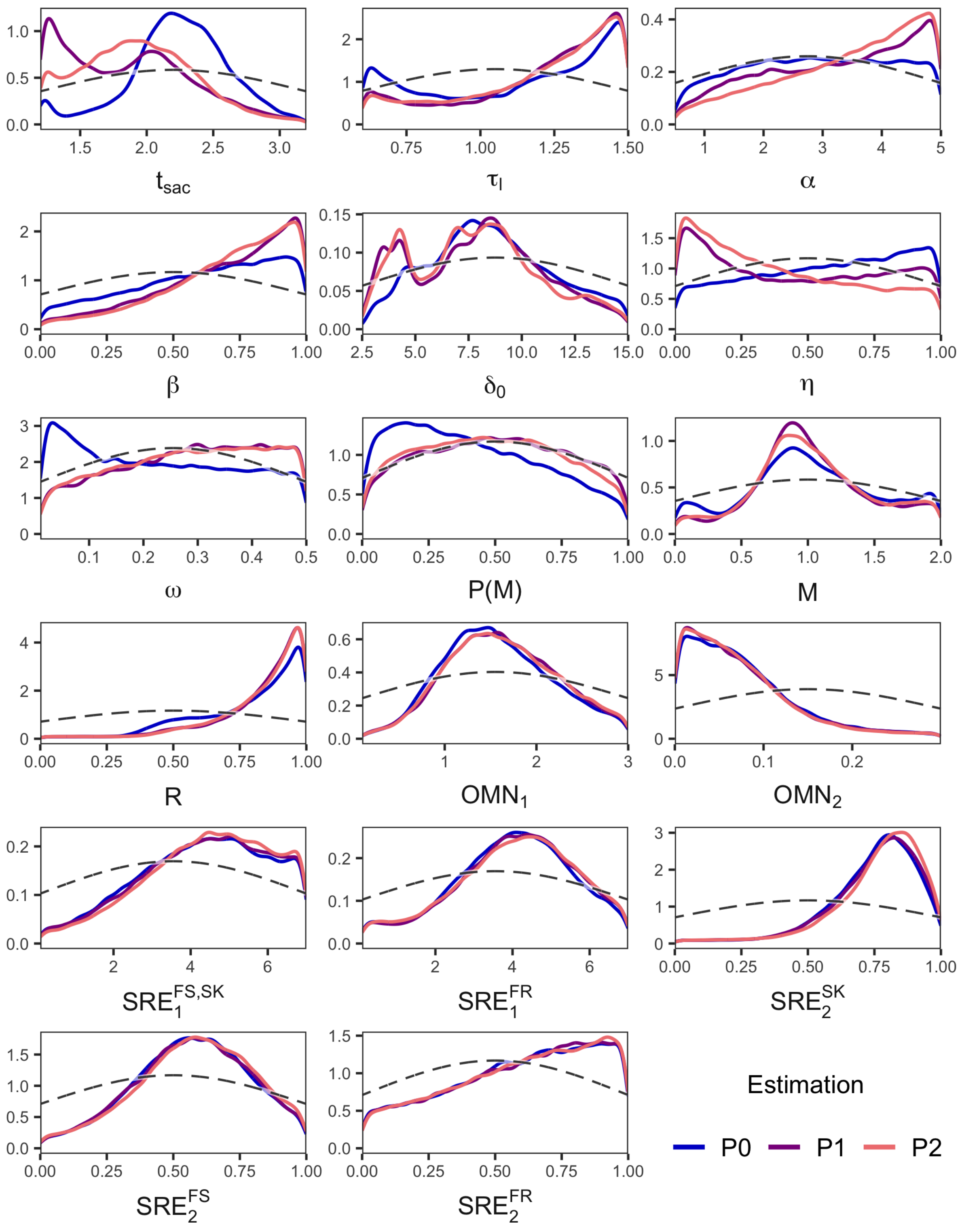

Figure S1. Posterior densities for all estimated parameters averaged over participants. Priors (truncated Gaussians) are given by dashed lines. The posteriors for the different model variants are indicated by different colors.

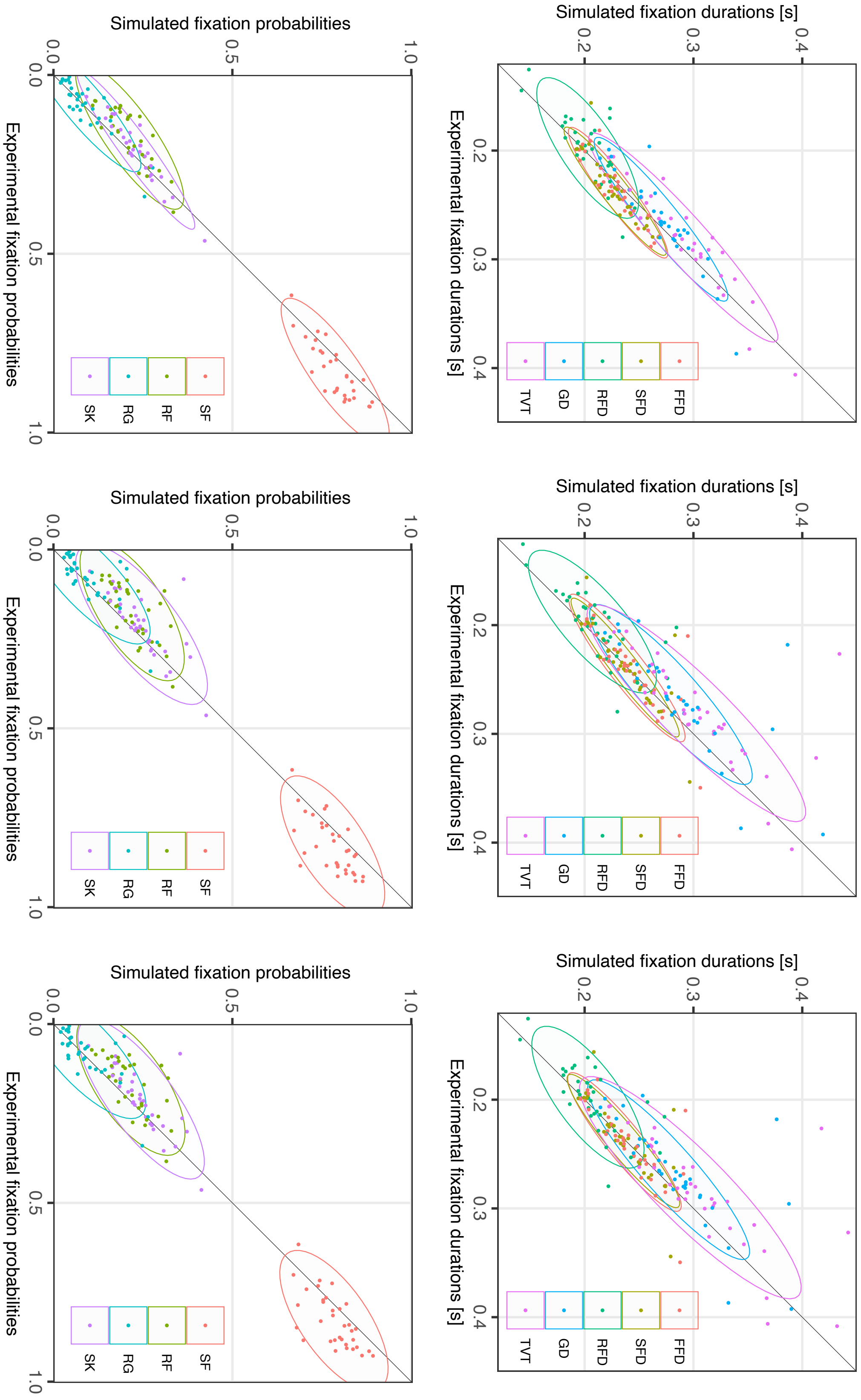

Figure S2. Correlation between common summary statistics for experimental and simulated data in the P0 dataset without saccade cancelation (baseline model). In all plots each point represents the means of experimental and simulated measure for a single participant.

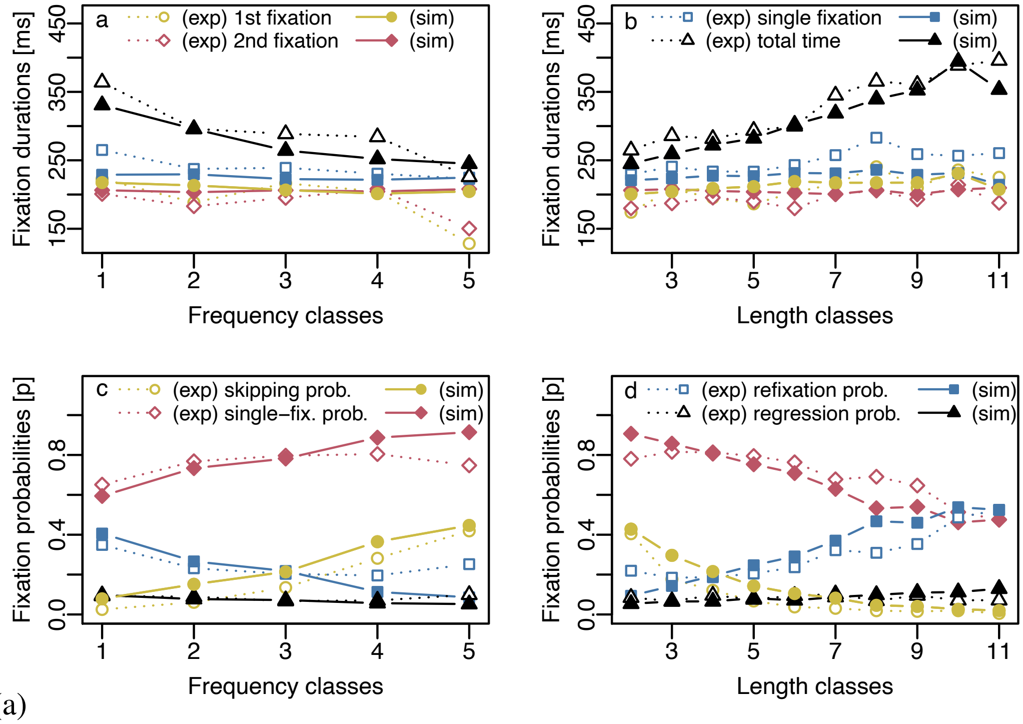

(a)

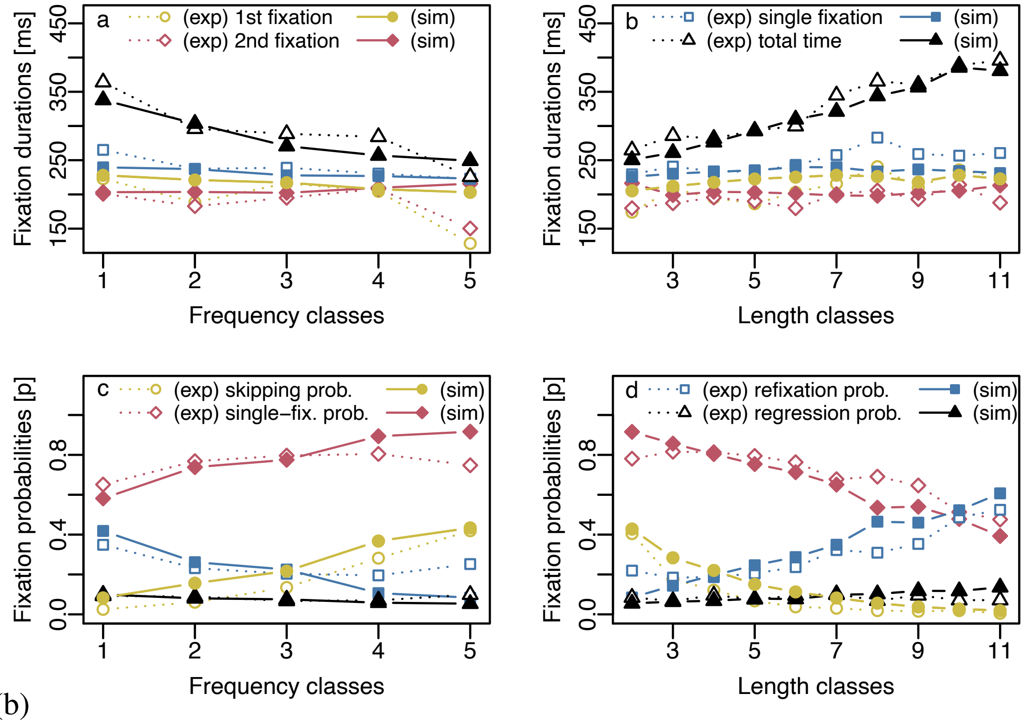

(b)

Figure S3. Summary statistics for experimental data (exp) and simulations (sim) of the control condition (with MF previews) for SWIFT models P0 (a), P1 (b) and P2 (c). Different measures of fixation durations and fixation probabilities are shown in the respective upper and lower panels. All measures were calculated for classes of word frequency (left panels) and and word length (right panels).

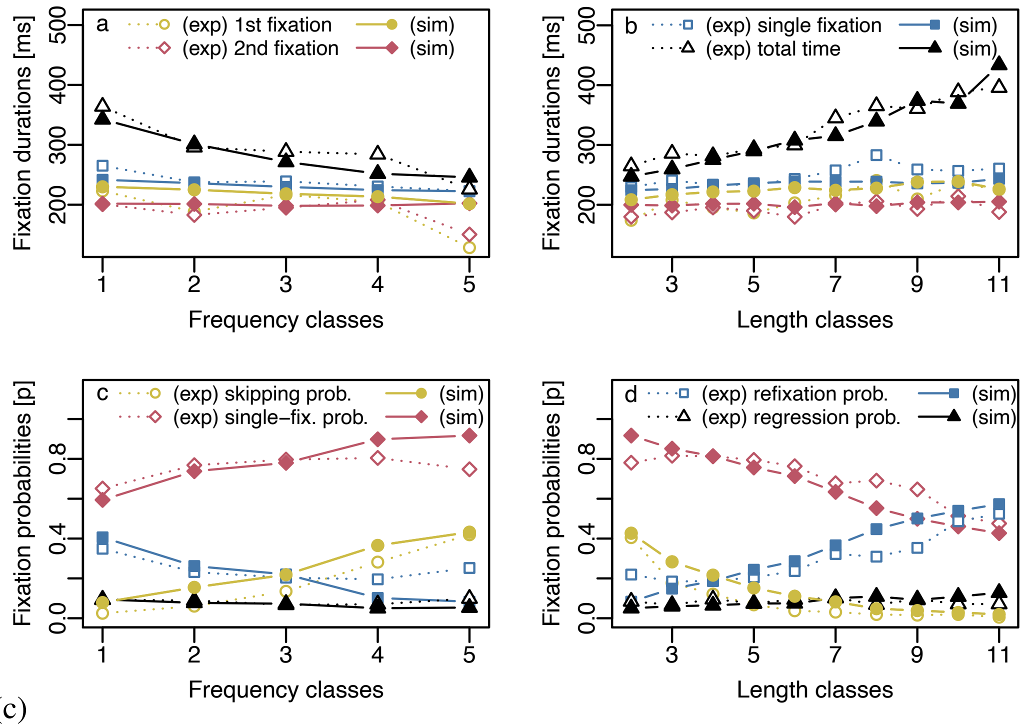

(c)

Figure S3 (continued).

Table S1. Parameters estimated for the three model variants. The range values indicate the boundaries of the Gaussian prior distributions which were symmetrically truncated at ±1 standard deviation.

		Range		P0			P1			P2		
Parameter	Symbol	lower	upper	min	HPDI (sd)	max	min	HPDI (sd)	max	min	HPDI (sd)	max
Saccade timer	τ_{sac}	120	320	213	224 (30)	239	159	187 (35)	206	175	195 (30)	210
Processing span	δ_0	2.5	15.0	7.28	8.35 (2.32)	9.52	6.79	7.7 (2.48)	9.15	6.68	7.68 (2.54)	8.96
Decay of activation	ω	0.01	0.5	0.15	0.22 (0.05)	0.3	0.23	0.29 (0.04)	0.36	0.22	0.29 (0.03)	0.35
Lexical difficulty: Slope	β	0.0	1.0	0.5	0.63 (0.1)	0.75	0.62	0.71 (0.12)	0.83	0.64	0.73 (0.1)	0.83
Lexical difficulty: Intercept	α	0.5	5.0	2.3	2.87 (0.61)	3.53	2.69	3.26 (0.67)	3.97	3.05	3.51 (0.62)	4.13
Word length exponent	η	0.0	1.0	0.43	0.58 (0.13)	0.72	0.26	0.46 (0.19)	0.62	0.22	0.37 (0.15)	0.5
Mislocated fixation probability	$P(M)$	0.0	1.0	0.27	0.41 (0.15)	0.52	0.37	0.51 (0.15)	0.63	0.35	0.49 (0.14)	0.61
Mislocated fixation factor	M	0.0	2.0	0.83	1.01 (0.32)	1.2	0.85	1.01 (0.25)	1.14	0.85	1.02 (0.26)	1.16
Labile saccade program	τ_{lab}	60	150	1.02	1.14 (0.24)	1.32	1.16	1.22 (0.2)	1.35	1.14	1.21 (0.19)	1.35
Refixation factor	R	0.0	1.0	0.75	0.79 (0.15)	0.91	0.82	0.84 (0.13)	0.93	0.81	0.84 (0.12)	0.93
Oculomotor noise: Intercept	omn_1	0.1	3.0	1.3	1.56 (0.36)	1.78	1.36	1.61 (0.34)	1.85	1.36	1.61 (0.34)	1.85
Oculomotor noise: Slope	omn_2	0.0	0.3	0.04	0.07 (0.04)	0.09	0.04	0.07 (0.04)	0.09	0.04	0.07 (0.04)	0.08
SRE: Intercept refixations	sre_1^{RF}	0.1	7.0	3.46	3.99 (1.04)	4.65	3.56	4.08 (1.03)	4.76	3.54	4.07 (1.05)	4.78
SRE: Slope refixations	sre_2^{RF}	0.0	1.0	0.49	0.61 (0.14)	0.74	0.49	0.62 (0.13)	0.75	0.5	0.61 (0.13)	0.75
SRE: Intercept forw. and skip.	$sre_1^{FS,SK}$	0.1	7.0	3.66	4.34 (1.12)	5.09	3.74	4.39 (1.06)	5.15	3.86	4.5 (1)	5.23
SRE: Slope forward saccades	sre_2^{FS}	0.0	1.0	0.49	0.57 (0.12)	0.66	0.49	0.57 (0.12)	0.66	0.5	0.58 (0.12)	0.68
SRE: Slope skipping saccades	sre_2^{SK}	0.0	1.0	0.71	0.74 (0.12)	0.82	0.72	0.75 (0.12)	0.83	0.74	0.77 (0.12)	0.85

Table S2. Model variants. Fixed parameter values were used for the three different implementations of foveal and parafoveal inhibition.

Model variant	Inhibition parameter foveal	Inhibition parameter parafoveal
No parafoveal inhibition (P0)	2.0	0.0
Parafoveal inhibition (P1)	2.0	3.0
Delayed parafoveal inhibition (P2)	2.0	3.0

Table S3. Pearson correlation coefficients for correlations between experimental and simulated mean fixation durations and fixation probabilities across participants for all estimations and conditions.

Summary statistics	P0			P1			P2		
	HF	MF	LF	HF	MF	LF	HF	MF	LF
**Fixation durations**									
Single Fixation Duration	.67	.71	.72	.78	.84	.77	.74	.80	.76
First Fixation Duration	.81	.81	.76	.70	.74	.67	.69	.72	.66
Re-Fixation Duration	.49*	.74	.49*	.50*	.74	.56	.52	.71	.55
Gaze Duration	.68	.72	.73	.77	.81	.76	.74	.78	.77
Total Viewing Time	.78	.81	.76	.72	.75	.73	.68	.73	.70
**Fixation probabilities**									
Single Fixation	.73	.76	.74	.73	.76	.75	.74	.78	.74
Refixation	.71	.74	.68	.69	.68	.68	.67	.68	.66
Regression	.79	.74	.76	.81	.80	.80	.76	.71	.75
Skipping	.71	.74	.68	.69	.68	.68	.67	.68	.66

* $p < .01$; all other values $p < .001$
